# Can Single Incision Laproscopic Cholecystectomy Replace the Traditional Four Port Laproscopic Approach: A Review

**DOI:** 10.5539/gjhs.v6n6p119

**Published:** 2014-07-15

**Authors:** Muhammad Umer Ahmed, Azib Aftab, Haseeb Munaf Seriwala, Ali Mahmood Khan, Khurram Anis, Iqbal Ahmed, Shafiq Ur Rehman

**Affiliations:** 1 Ziauddin University, Ziauddin Medical College, Karachi, Pakistan; 2 Aga Khan University Hospital, Karachi, Pakistan; 3 Dow University of Health Sciences (DUHS), Karachi, Pakistan; 4 Milton Keynes General Hospital, United Kingdom; 5 Department of Surgery Ziauddin University Hospital, Pakistan; 6 Head of Department of Surgery, Ziauddin University Hospital, Pakistan

**Keywords:** SILS: Single Incision Laparoscopic Surgery, SILC: Single Incision Laparoscopic Cholecystectomy, 4PLC: 4 Port Laparoscopic Cholecystectomy, NOTES: Natural Orifice Transluminal Endoscopic Surgery, LESS: Laparoendoscopic Single-Site Surgery

## Abstract

The major aim of surgeons has always been a minimalist approach towards surgery, thereby reducing the complications associated with the surgery. The gold standard treatment for cholelithiasis with cholecystitis is currently the four port laparoscopic cholecystectomy (4 PLC). Recently, a newer technique has been introduced which uses a single port, rather than the four ports, for the removal of the gall bladder laparoscopically; it is known as Single Incision Laparoscopic Cholecystectomy (SILC). This is a comparatively minimal approach towards surgery. Therefore the purpose of this review is to compare the advantages and the disadvantages of SILC versus 4PLC, and hence, to give an idea of whether SILC is ready to replace the traditional approach as the new treatment of choice.

## 1. Introduction

Surgery has always been one of the feared treatment options for most of the patients; therefore, it has always been the ultimate goal of surgeons all around the world to provide the patients with the best possible surgical option. The best possible surgical option has always been the one with the minimum number of complications postoperatively, along with well-controlled pain and minimum stay at hospital. In the busy world that exists currently, no one likes a prolonged stay in the hospital, both prior to surgery and postoperatively. Also, extended hospital stay has been associated with increased incidence of hospital acquired infections, which causes further increase in mortality (Kaye et al., 2014). Therefore, to facilitate the needs and comfort of patients is equally important as compared to maintaining the quality of the surgery and its outcome.

The first surgical intervention for gall bladder was an elective surgery carried out by Langenbuch in 1882 (Soper, 2011) via an open approach. Open cholecystectomy became increasingly popular in the 1980s with less than 1% of mortality rates and an even lower incidence of bile duct injury with morbidity of 0.1-0.2% (Morgenstern, Wong, & Berci, 1992; Roslyn et al., 1993). This traditional procedure was although performed through a larger abdominal incision, and therefore, had significant associated pain as well as a lengthier recovery period, requiring a longer stay in hospital.

Laparoscopic surgery was initially introduced in the 1980s, with the first laparoscopic surgery being performed by Mühe (Mühe, 1991). His approach was soon followed by the surgeons in France and America. This made this new technique a plausible replacement for the traditional open approach; hence, soon it became the standard of care. The approach became very popular among the patients since it resulted in a shorter hospital stay, minimal scars and less pain (Moreira-Pinto, Lima, Correia-Pinto, & Rolanda, 2011). Therefore, invention of minimally invasive surgery via laparoscopicapproach for the gall bladder, also opened doors to minimalist approach at the structures related to gall bladder, and soon techniques like cholangiography via laparoscopy, CBD (Common Bile Duct) exploration and choledocotomy which came into routine use for the treatment of biliary disease (Antoniou, Pointner, & Granderath, 2011), giving the biliary tract a new gold standard treatment in the face of minimally invasive surgery. The increasing preference shown by both surgeons and patients for laparoscopic approach over the traditional open cholecystectomy clearly shows the demand of the world towards as minimalist of a surgical approach as possible.

The enthusiasm with which laparoscopic surgery was accepted by the doctors and patients was tremendous and hence, it paved way for the once deemed impossible incision-less surgery. In 2004, revolution was seen in the form of the first transgastric peritoneoscopy, thus giving light to the idea of Natural Orifice Transluminal Endoscopic Surgery (NOTES). Incision-less surgery therefore became the new goal for surgeons around the world (Moreira-Pinto et al., 2011). The importance of an incision less surgery cannot be stressed enough when considering the advantages that it has to offer to the surgeons and the patients. NOTES not only eliminates the need for an incision, but it also decreases the post-operative pain (reducing the need for the analgesics to a bare minimum and ensuring patient comfort and satisfaction). NOTES allows the procedures to be performed on an outpatient based setting, which not only reduces the burden on tertiary care hospitals, but also makes this a suitable option for the patients who do not wish to stay in the hospital for long. This causes a decrease in the rate of ignorance of disease and the mortality rate associated with cholelithiasis. Along with these advantages, there has been a marked reduction in the incidence of post-surgery hernias and therefore a greater increase in patient’s satisfaction (Moreira-Pinto et al., 2011; Shafi, Mery, Binyamin, & Dutta, 2006). But, NOTES is not as fruitful as it sounds. It has an associated concern of the inability to find a clean site for access, thereby increasing the chances of intra-abdominal spillage or infection from the incision (Shafi et al., 2006). Other than this, there is a lack of a single technique to close the luminal incision for the various access sites, thereby limiting the application of this technique. Moreover, the need for pneumo-peritoneum, for adequate visualization, has been associated with bowel over distention and is still a concern, as it is for the traditional laparoscopic biliary surgery (Moreira-Pinto et al., 2011). Therefore, with the current ongoing research and the available literature review, is still not sufficient to entitle NOTES as the replacement of traditional surgery of the biliary tract via laparoscopic approach.

Among the current surgical developments, a notable development is the SILS (Single Incision Laparoscopic Surgery). SILS was recommended as a possible alternative of the traditional laparoscopic surgery via four ports for the biliary tact by Navarra et al. (Navarra, Pozza, Occhionorelli, Carcoforo, & Donini, 1997). With NOTES having a diminished success, increased interest has been seen in SILS. Hence, LESS (Laparo endoscopic single site surgery) came into being. These complementary revelations have similar problems associated with access and approach during surgery. But, LESS is still a developing technique with more to prove before becoming gold standard. Currently it occupies a space between NOTES and standard laproscopy (Prashanth, Pradeep, & Bhagwat, 2011). Other than being minimally invasive, it remains the goal of laparoscopic cholecystectomy by single incision (SILC) to provide the patients with a more satisfactory and comfortable alternate in terms of decreased length of hospital stay, decreased pain and better aesthetic results (Antoniou et al., 2011; Gill et al., 2010). There has been some of research and citation related to adoption of SILC/LESS as the possible replacement of traditional four port cholecystectomy including the statement of consensus in 2010 by the Laparoendoscopic Single-Site Surgery Consortium for Assessment and Research (LESSCAR) (Gill et al., 2010). Therefore, the purpose of this article is to review and assess the results related to the SILC/LESS to a certain whether this can replace as the gold standard treatment of choice.

## 2. Discussion

The field of minimally invasive surgery has become a major focus throughout the entire surgical world, gallbladder removal being no exception to this. The replacement of the conventional 4 Port Laparoscopic Cholecystectomy (4PLC) by Single Incision Laparoscopic Cholecystectomy (SILC) as the most utilized surgery procedure or gold standard (Chamberlain & Sakpal, 2009) is currently under scrutiny. A comparison of both techniques has a need to be looked into.

### 2.1 Cosmetic

One of the greatest advantages of SILC over 4PLC is the reduction in incisions needed. Where in SILC there is only need for one 12-20 mm umbilical skin incision (Fransen, Stassen, & Bouvy, 2012), in 4PLC there is need for four incisions, two 5 mm ports and two 10-mm ports (Gurusamy, Vaughan, Rossi, & Davidson, 2014). In a study of scar assessment left by both 4PLC and SILC by Ostlie et al. (Ostlie et al., 2013a), it was concluded that there is a positive response at long-term follow-up. SILC has been said to have significant long-term cosmetic benefit in several other studies as well (Milas, Deveđija, & Trkulja, 2014; Marks et al., 2011; Lirici, Califano, Angelini, & Corcione, 2011; Bucher, Pugin, Buchs, Ostermann, & Morel, 2011). Early cosmetic benefit was modest though (Ostlie et al., 2013a; Milas et al., 2014). These studies were conducted using body image scales, a 10 point scale used by Mark et al. and Butcher et al. while a four point scale was used by Ostlie et al., in an attempt to reduce bias in the evaluation. According to these studies, patients were more satisfied with the cosmetic appearance of SILC over 4PLC.

### 2.2 Improvement of Operative Time

As SILC is considered to be a relatively unused technique, it is imperative to understand the learning experience of the surgeons and how the operative time changes with experience. In a study conducted by Tay et al. (2013) which was focused on the learning curve of SILC two surgeons were observed to see if their mean operating time reduced with experience. In the first surgeon, over a period of 97 operations, the operation time fell from more than 90 minutes to under 37 minutes. This evidence is also supported by Qui et al. study (2011) in which the average time of operation time was 46.9 minutes, but after 40 cases they succeeded in bring the mean operative time to under 40 minutes. Hernandez et al. (2010) also found a drastic reduction in operative times after completion of 75 SILC procedures by a single surgeon. This fact is also supported by Duron et al. (Duron, Nicastri, & Gill, 2011). Interestingly though, a study conducted by Mutter et al., to see the implementation in a teaching hospital, stated that even though the surgeons with experience performed more rapidly, there was no significant difference (Mutter et al., 2011). But this can be downplayed by the studyconducted by Tay et al. (2013) in which a second surgeon showed faster improvement on mentoring from the first surgeon. Also, when having an assistant with 4PLC experience, the average operative time for a SILC operation was 48 minutes as opposed to the 74 minutes for having and assistant without any experience. Joseph et al. (Joseph, Phillips, & Rupp, 2012) also noted a reduction in operative time.Overall, the learning curve is said to be quite short (Solomon, Bell, Duffy, & Roberts, 2010). Hence, with experience, the time required for the operation drastically decreases.

### 2.3 Prolonged Time of Surgery

An important comparison between both cholecystectomy procedures is the actual time of the surgery which is one of the most tangible results in studies of both procedures. In a research by Ma et al. (Ma et al., 2011), where the patients for SILC (n = 21) and 4PLC (n = 22) were almost the same, the average operative time was 88.5 minutes for SILC and 44.8 minutes for 4PLC which is almost double in difference. This is supported in other studies as well (Marks et al., 2011; Lirici et al., 2011; Bucher et al., 2011; Phillips et al., 2012; Gangl, Hofer, Tomaselli, Sautner, & Függer, 2011; Lee et al., 2010). According to Gangl et al. (Gangl et al., 2011), which had more patients, there was only a difference in SILC and 4PLC times of 75 minutes to 63 minutes respectively. The reduction in the difference could be due to improvement in operative time due to experience as related by Tay et al. (2013). This is supported by Milas et al. (2014). Most trials have a small number of patients which undergo SILC so it is argued that results from these studies are incomplete (Tay et al., 2013). It is also stated that experienced doctors of SILC will have a lower mean operative time. On the other hand, the study by Phillips et al. (2012) shows that even with a large number of SILC operations (n = 117) the operative time for SILC is still longer that 4PLC. Thus, it is still inconclusive as to whether SILC is a faster or slower operation as compared to 4PLC.

### 2.4 Techinical Difficulty

The use of single port surgery is said to pose many technical difficulties (Gill et al., 2010). Diverse types of instruments are needed in this procedure such as cured and articulate instruments according to what the surgeon prefers (Phillips et al., 2012). Also, using left and right-handed instruments which show up on opposite sides of the screen increase the difficulty (Antoniou et al., 2011). In addition, instruments with articulated handles as well as variable lengths are used (Romanelli & Earle, 2009). This helps avoid collisions as well as allow wider range of movement with the instruments. The use of a wide variety and complexity of instruments and tools leads to amplified technical difficulty (Ostlie et al., 2013b). It can be said that SILC outcomes largely depend on the surgeon’s skill (Milas et al., 2014).

### 2.5 Increased Duration of General Anesthesia

One of the greater drawbacks of SILC when compared to 4PLC is the longer duration of general anesthesia given the patient. This is due to prolonged operative time, and as a result, there is increased patient risk. Most studies have used ASA classes III or IV as the stopping point for patients that were to undergo SILC. Thus, there haven’t been any anesthesia related complications (Marks et al., 2011; Lirici et al., 2011; Phillips et al., 2012; Roberts, Solomon, Duffy, & Bell, 2010; Tsimoyiannis et al., 2010). Inconclusive research has been done based on all ASA classes, and as such, and problems arising from anesthesia have not been properly checked.

### 2.6 Complications

While in a survey conducted by Rao et al. (Rao, Kynaston, MacDonald, & Ahmed, 2010), SILC was accepted by 34.3% of patients without any safety profile, it still remains to be seen whether or not the procedure is safe enough to use as a standard. The intraoperative and postoperative complications must be reviewed. Most studies have reported no significance in complication rate (Antoniou et al., 2011; Milas et al., 2014; Marks et al., 2011; Bucher et al., 2011; Gangl et al., 2011; Lee et al., 2010; Roberts, Solomon, Duffy, & Bell, 2010; Lai et al., 2011). Some though, have indicated a significant increase in the complication rate of SILC (Ma et al., 2011; Phillips et al., 2012). Many though, are inconclusive and suggest that without large randomized trails, the safety of this procedure cannot be gauged (Franserl et al., 2012; Gurusamy et al., 2014; Lirici et al., 2011).

In terms of successful surgeries, Antoniou et al. (2011) analyzed 1166 patients from 29 different articles and reported a 9.3% unsuccessful surgery rate when using the SILC method. This was mainly due to improper identification of the Calot’s triangle. In a study conducted by Yeo et al. (Yeo, Mackay, & Martin, 2012), intraoperative cholangiography (IOC) and exploration of common bile duct was used as needed through the umbilical port. Out of 60 patients, 55 were successful and 53 of them received IOCs. The success rate was 91.7% meaning that the unsuccessful surgery rate was 9.3%, the same as Antoniou et al. (2011). The number of patients was small so no benefit in using IOCs could be seen.

Among some of the most common complications is post-incisional hernia which has been attempted to be eliminated by converting several fascial defects into a single incision (Antoniou et al., 2011; Phillips et al., 2012; Romanelli & Earle, 2009). There were complications of wound infections, incisional hernia and bile leakage in Yeo et al.’s study (Yeo et al., 2012). In a review of complications in SILC by Fransen et al. over 1180 patients in 38 articles (Franserl et al., 2012), there were 24 cases of minor injuries (2%), and 32 cases of major complications (2.7%). Most major complications were due to bile duct lesions, retained stones and readmission due to pain. This is supported by the findings of Antoniou et al. (Antoniou et al., 2011).The only difference was that Antoniou et al. reported that patients older than 45 years were more prone to complications. Overall though, Fransen et al. (2012) deduced that these results were not much different from results of 4PLC. In addition, it was stated that lack of experience was also a factor in causing complications, indicating that the SILC procedure would have less complications when in full use.

### 2.7 Post-Operative Pain

In order for patients to return back to their daily activities, postoperative pain needs to be less. Less post-operative pain also allows less use of analgesics. Gangl et al. (2011) conducted a research in which they assessed pain 24 hours after the operation and 48 hours after as well. A visual analogous scale was used, and the findings suggested that there was not any significant difference between post-operative pain in both procedures. On the other hand, many studies showed a significant increase in post-operative pain (Marks et al., 2011; Lirici et al., 2011; Ma et al., 2011; Phillips et al., 2012; Lee et al., 2010; Ostlie et al., 2013b; Lai et al., 2011) for SILC. Interestingly though, there are studies that indicate less pain in SILC as well (Bucher et al., 2011; Tsimoyiannis et al., 2010). According to Tsimoyiannis et al. the major difference in pain between SILC and 4PLC is the significantly lower shoulder pain and abdominal scores, especially 24 hours after the operation (Tsimoyiannis et al., 2010). This was evaluated using the visual analogue scale at 2, 6, 12, 24, 48 and 72 hours post-operation. As pain is felt differently for each patient, it is difficult to conclude on whether or not there is less post-operative pain. More study is required in this area.

### 2.8 Length of Hospital Stay

Cutting down the time spent in the hospital is beneficial to both hospital and patient, as it reduces costs. SILC was said to have a significantly shorter stay in the hospital by Lee et al. (2010). This was supported by a few other studies (Moreira-Pinto et al., 2011; Antoniou et al., 2011; Gill et al., 2010). Many though, didn’t find a much significant difference in hospital stay (Bucher et al., 2011; Gangl et al., 2011; Lai et al., 2011). SILC is regarded to have a shorter hospital stay than normal 4PLC in most cases.

### 2.9 Cost

In a comparison of costs, it is said that SILC is more expensive (Bucher et al., 2011; Ostlie et al., 2013b; Bucher et al., 2009). This was in spite of Bucher et al. (2009) reusing material in an attempt to reduce costs. In a study by Love et al. (2011) though, specifically reviewed the cost comparison, and it was concluded there was not any significant difference in cost when standard materials and equipment were used and the duration of the procedure considered (Love et al., 2011). This is said to be due to the products required in the operation being under development (Ostlie et al., 2013b), and that these costs cannot be compared to those costs of an operation being done routinely. Hence, with increased usage of the SILC procedure, the costs may reduce.

## 3. Conclusion

Ever since the first SILC in 1997 by Navarra et al. (1997), this minimally invasive procedure has been compared more and more to the normal method of 4PLC. While the cosmetic result of SILC is appreciated over that of 4PLC, SILC has yet to become the gold-standard procedure for surgical removal of gallbladder. This technically difficult (Milas et al., 2014) method has not been available to a large variety of patients. According to current evidence, this process is costly although this may change as SILC is used more often It is inconclusive as to whether SILC is faster or slower in operative time as compared to 4PLC, due to the reported decrease in mean operative time as experience is gained (Yeo et al., 2012), whereas others reported higher mean operative times. Also, due to the lack of a large number of randomized trials the complications associated with this surgical method cannot be fully comprehended. Patient safety hasn’t been confirmed in SILC nor has there been a clear indication of less post-operative pain after SILC because of the difficulty in measuring pain. Standardization and further randomized trials are required for surgeons around the world to verify whether or not SILC can substitute 4PLC. The increased reports will help in arriving to a verdict about certain areas which are currently inconclusive. Hence, SILC is a procedure still in the progress of being established in the surgical field of minimally invasive surgery.
